# A Case of Overlapping Choriocapillaritis Syndromes: Multimodal Imaging Appraisal

**Published:** 2012-01

**Authors:** Tatiana Kuznetcova, Bruno Jeannin, Carl P Herbort

**Affiliations:** 1 Center for Ophthalmic Specialized Care, Lausanne, Switzerland; 2 Department of Ophthalmology, I.P. Pavlov State Medical University, Saint-Petersburg, Russia; 3 University of Lausanne, Lausanne, Switzerland

**Keywords:** Choriocapillaritis, Multiple Evanescent White Dot Syndrome, Multifocal Choroiditis Indocyanine Green Angiography, Optical Coherence Tomography, Fundus Autofluorescence

## Abstract

**Purpose:**

To present a patient with overlapping choriocapillaritis syndromes who first presented as a typical case of multiple evanescent white dot syndrome (MEWDS) and later with characteristic findings compatible with multifocal choroiditis (MFC).

**Case Report:**

A 40-year-old myopic woman presented with a paracentral scotoma OS. Fundus examination revealed pale discolored areas around the optic disc corresponding to faintly hyperfluorescent areas on fluorescein angiography (FA). On indocyanine green angiography (ICGA) there was extensive peripapillary hypofluorescence and confluent hypofluorescent dots superiorly. According to the clinical picture, a diagnosis of MEWDS was made. In 4 weeks, the visual field reverted to normal together with almost complete regression of hypofluorescence on ICGA. However, 4 months later fundus examination revealed some scars, a finding not typical for MEWDS. Besides, she developed another scotoma 12 months later accompanied by photopsia and the fundus illustrated more numerous scars than one year earlier. ICGA showed hypofluorescent areas corresponding to the scotoma delineated by visual field testing. The pattern of this recurrence clearly corresponded to MFC.

**Conclusion:**

This case illustrates an overlap between two entities, MEWDS and MFC in two sequential episodes. FA and fundus autofluorescence accounted for the lesions and optical coherence tomography showed damage to the photoreceptor outer segments, but only ICGA correlated well with functional evolution.

## INTRODUCTION

Primary inflammatory choriocapillaropathies (PICCPs) are a group of disorders characterized by choriocapillaritis causing sectoral nonperfusion of the choriocapillaris.^1^ They comprise most of the diseases formerly classified within the umbrella term “white dot syndromes”,^2^ including multiple evanescent white dot syndrome (MEWDS), acute posterior multifocal placoid pigment epitheliopathy (APMPPE), multifocal choroiditis (MFC), serpiginous choroiditis (SC) and some rare entities. The most appropriate technique to investigate these entities is indocyanine green angiography (ICGA) which shows diverse patterns of hypofluorescence indicating choriocapillaris hypo or non-perfusion which is the common denominator of these diseases.^3^–^6^ The disease mechanism had already been suspected well before the availability of ICGA and findings based on this imaging modality only confirmed the suspected pathophysiology for this group of disorders.^7^ The consequence of choriocapillaris non-perfusion is outer retinal ischemia with damage to the photoreceptor outer segment layer (POSL).^8^ The extent of hypofluorescent non-perfusion and the degree of associated ischemia determine the severity of each entity; MEWDS is on the benign end of the spectrum while serpiginous choroiditis which entails irreversible chorioretinal atrophy lies at the more severe end.^9^ Intermediate forms of choroicapillaritis such as ampiginous choroiditis or relentless placoid choroiditis have been described with characteristics of APMPPE and serpiginous choroiditis (SC).^10^,^11^ More recently, choriocapillaritis with positive interferon-gamma release assays which manifests with mixed features of APMPPE and SC has been termed multifocal-serpiginous choroiditis.^12^,^13^ Unclassifiable forms that cannot be categorized under one of the well-determined entities have also been reported.^14^ Moreover, several cases have been published in which the patient presented with one choriocapillaritis syndrome at a time but with features consistent with another entity at another time.^15^–^18^ Herein, we present a case of overlapping choriocapillaritis syndromes; the patient was first diagnosed with MEWDS but demonstrated typical findings corresponding to multifocal choroiditis a year later. Analysis of the morphologic and pathophysiologic features of both inflammatory episodes could be achieved thanks to multimodal imaging including fundus examination/photography, fluorescein angiography (FA), ICGA, fundus autofluorescence (FAF) and optical coherence tomography (OCT). Functional parameters including visual field testing and microperimetry facilitated the diagnosis and management.

## CASE REPORT

A 40-year-old myopic female presented with a paracentral scotoma OS of about one week’s duration. Her past ocular history revealed high bilateral myopia of 11.75 diopters (D) in the right eye (OD) and 10.0 D in the left eye (OS). She had been operated for rhegmatogenous retinal detachment in her right eye 15 years earlier and 360 degrees circumferential prophylactic argon laser treatment had been performed for both eyes. Two years before the current episode, cataract surgery had been performed followed by Nd:YAG laser capsulotomy in both eyes a few months later. To treat residual astigmatism, an arcuate corneal incision had been performed in the left eye a few months following cataract surgery.

**Figure 1.**
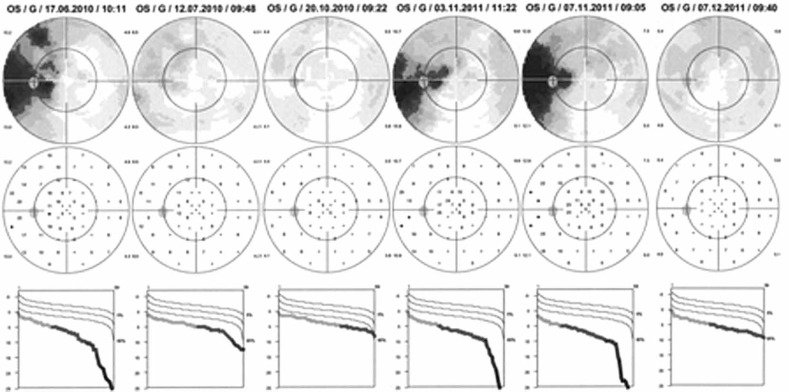
Evolution of the visual field defect during the first episode of choriocapillaritis diagnosed as multiple evanescent white dot syndrome (MEWDS) and the second episode characterized as multifocal choroiditis. The peripapillary defect at presentation (left column) corresponded to indocyanine green angiography (ICGA) hypofluorescence (figure 4) and resolved without treatment (second and third columns) after the first choriocapillaritis episode diagnosed as MEWDS. Sixteen months later, a similar visual field defect again corresponding to ICGA hypofluorescence (figure 7) developed during a second episode of choriocapillaritis (fourth column) and progressed over a period of 4 days (second column from the right) but resolved (right column) one month after a sub-Tenon’s injection of triamcinolone in parallel with resolution of ICGA hypofluorescence.

Upon presentation, best corrected visual acuity (BCVA) was 0.9 and 0.8 in the right and left eyes, respectively. No signs of inflammation were detected in the anterior chambers, but laser flare photometry (LFP) values were slightly elevated; up to 9.5 ph/ms OD and 13.2 ph/ms OS (normal values: 3–6 ph/ms). There were no cells visible in the vitreous. Visual field testing using the Octopus Perimeter® (Haag-Streit, Bern, Switzerland) showed a peripapillary scotoma OS (Fig. 1) and a normal visual field OD. Microperimetry displayed slightly diminished retinal sensitivity OS with a test score of 390/560 versus 414/560 OD.

**Figure 2.**
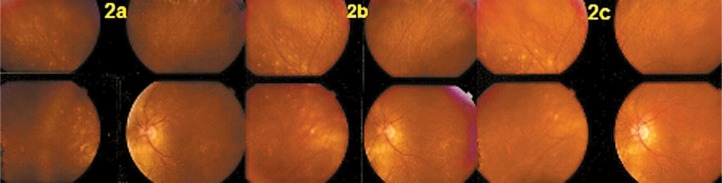
Fundus images at presentation (2a), after 1 month (2b), and at 4 months (2c). Faint discolored peripapillary fundus lesions typical of multiple evanescent white dot syndrome (MEWDS) were seen at presentation (2a) and still present after one month (2b); corresponding to fluorescein angiography (FA) hyperfluorescent areas and hyperautofluorescent areas. At 4 months (2c), these lesions take the aspect of chorioretinal scars while FA and hyperautofluorescent lesions are no longer present.

**Figure 3.**
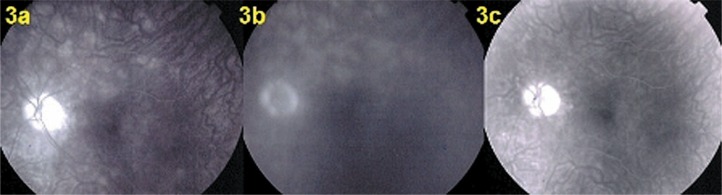
Fluorescein angiography at presentation (3a), after 1 month (3b), and at 4 months (3c). Patchy hyperfluorescent areas were seen at presentation around the optic disc and along the temporal superior arcade (3a) and were still present at 1 month (3b), but resolved after 4 months (3c).

**Figure 4.**
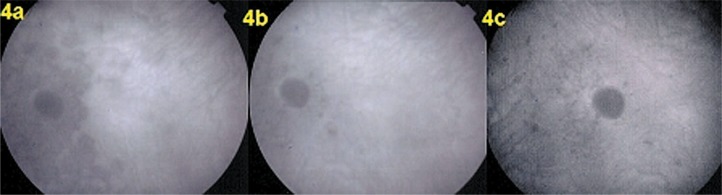
Indocyanine green angiography at presentation (4a), after 1 month (4b), and at 4 months (4c). **C**onfluent **p**eripapillary hypofluorescence and scattered hypofluorescent areas are seen superiorly at presentation (4a), which almost completely resolved after one month (4b). However at 4 months (4c), hypofluorescent pinpoints are seen indicating chorioretinal scars.

**Figure 5.**
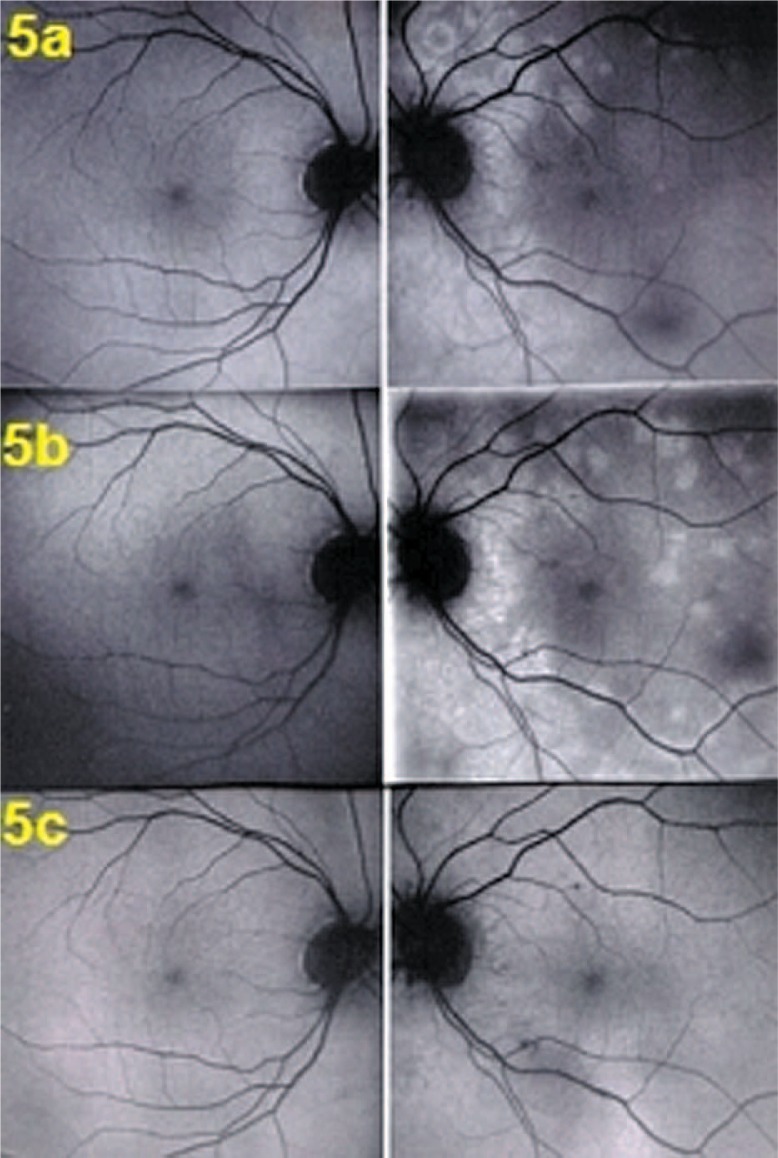
Fundus autofluorescence pictures at presentation (5a), after 1 month (5b), and at 4 months (5c). On the right column (left eye), hyper-autofluorescent areas corresponding to FA and ICGA lesions can be seen at presentation (5a) which, on the middle picture, slightly progressed after one month (5b) with return to normal at 4 months (5c).

**Figure 6.**
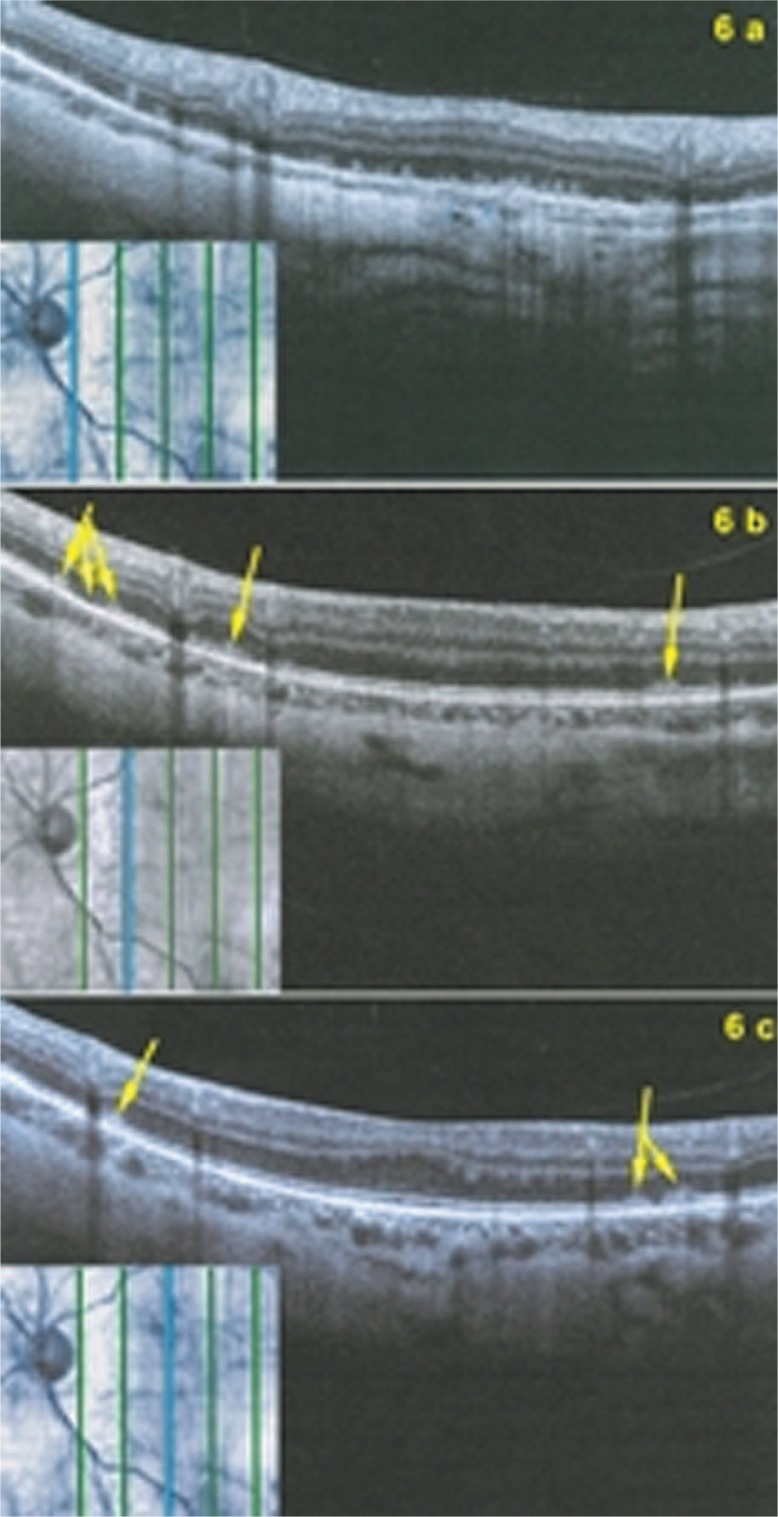
Optical coherence tomography scans at presentation through ICGA hypofluorescent areas (6a), and at the border of ICGA hypofluorescent area (6b and 6c). The top set of figures (6a) represents a scan going through the ICGA hypofluorescent area showing damaged photoreceptor outer segment layer (POSL) with clumps throughout the whole scan. 6b and 6c show scans at the border of the ICGA hypofluorescent demonstrating sectoral damage to POSL (arrows).

Fundus examination showed pale discolored dots around the optic disc and along the superior temporal arcade OS (Fig. 2a) which corresponded to faintly hyperfluorescent areas on FA (Fig. 3a). More precise information on the diseased areas was revealed by ICGA: there was extensive peripapillary hypofluorescence extending inferiorly together with centripetal hypofluorescent dots along the superior temporal arcade (Fig. 4a). These areas were precisely delineated by FAF, showing hyper-autofluorescence corresponding to ICGA hypofluorescent areas (Fig. 5a). Spectral domain OCT imaging (Heidelberg Instruments, Heidelberg, Germany) clearly showed POSL damage in scans through ICGA hypofluorescent and FAF hyper-autofluorescent areas, which also corresponded to the visual field defect. The whole POSL was damaged on scans through the ICGA hypofluorescent area (Fig. 6a; arrows), but damage was partial in bordering areas (Figures 6b and 6c; arrows).

Four weeks later, discolored lesions were still visible in the fundus (Fig. 2b), FA and FAF illustrated a few scattered new lesions (Figures 3b and 5b), but on ICGA the hypofluorescence had regressed almost completely and this was related with visual field recovery (Figures 4b and 1; second column from the left).

Four months later, fundus examination showed scars in areas where lesions had been present, a finding not typical of MEWDS (Fig. 2c). These scars were also seen on FA frames in the form of late hyperfluorescent dots (Fig. 3c) as well as on ICGA frames in the form of hypofluorescent dots (Fig. 4c). Along the superior temporal arcade, FA hyperfluorescent areas had disappeared (Fig. 3c) and on FAF a normal homogeneous aspect was seen (Fig. 5c).

**Figure 7.**
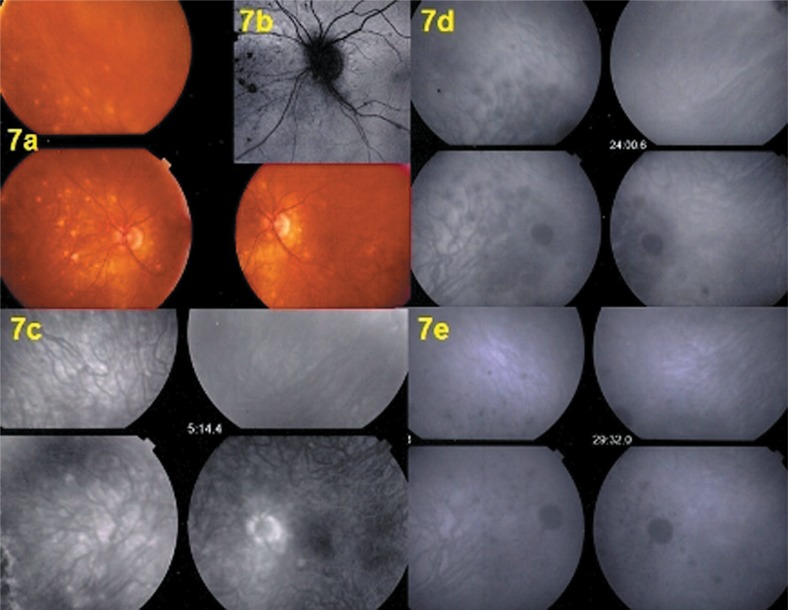
Second episode of choriocapillaritis consistent with multifocal choroiditis. Fundus images (7a) show chorioretinal scars nasal to the optic disc. Both fundus autofluorescence (7b) and fluorescein angiography (7c) show only faint lesions, while on indocyanine green angiography, hypofluorescence is substantial (7d), but resolves one month after periocular triamcinolone injection (7e) in parallel with recovery of the visual field (see figure 1).

**Figure 8.**
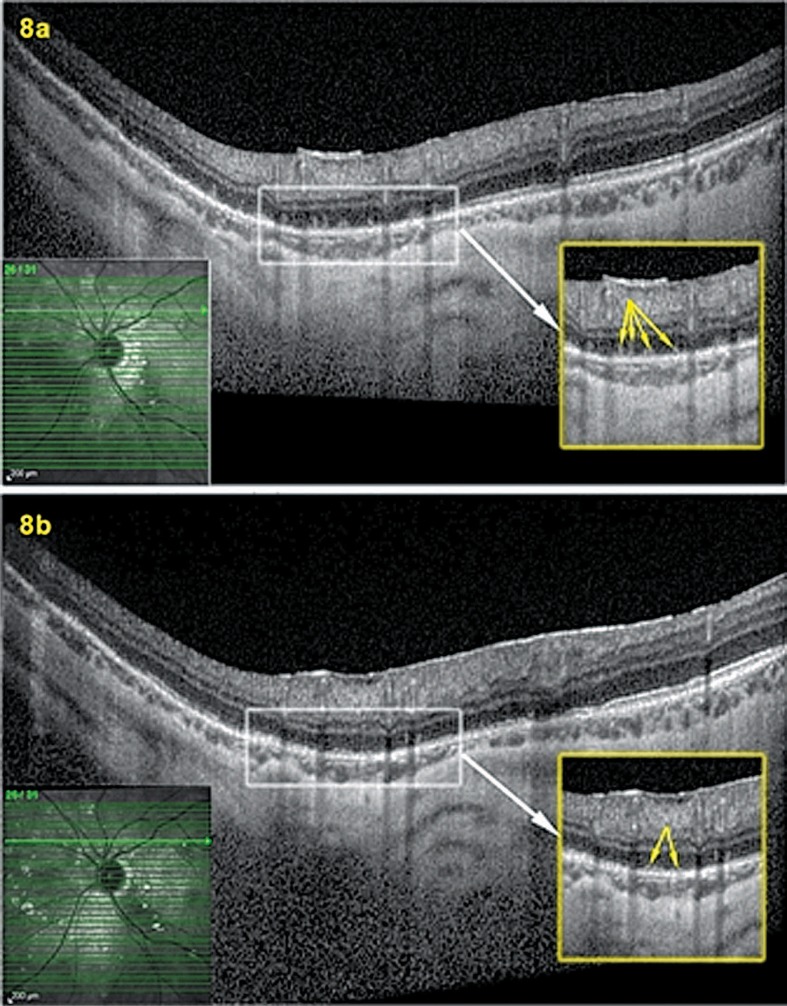
Spectral domain OCT showing scans going through ICGA hypofluorescent area during acute (8a) and convalescent (8b) stages of the disease. During the acute stage (8a) there is damage to the photoreceptor outer segment layer (POSL) showing clumps of photoreceptor outer segments (arrows) in ICGA hypofluorescent areas. During the convalescent stage (8b) regeneration of the POSL (arrows) is noted.

Twelve months after the last follow-up (16 months after the first episode), the patient was seen again for the same subjective scotoma in her left eye accompanied by photopsias. BCVA was 1.0 OD and 0.9 OS. No inflammation was noted in the anterior chamber or in the vitreous. LFP values were 6.0 ph/ms OD and 8.9 ph/ ms OS, showing slight subclinical flare OS. Intraocular pressure was 14 mmHg bilaterally. On fundus examination peripapillary and superior nasal scars were more numerous than 12 months earlier (Fig. 7a). Octopus® perimetry showed a peripapillary scotoma similar to the one seen during the prior presentation which increased 4 days later (Fig. 1; third and second columns from the right); the microperimetry score declined again to 338/560 as during the first episode. On FA there were less intensely hyperfluorescent zones indicating more atrophic areas than acutely involved retina (Fig. 7c). FAF did not show the intense hyper-autofluorescence seen during the first episode (Fig. 7b). Only ICGA accounted for the functional impairment recorded by visual field testing and microperimetry, and allowed to identify the involved areas showing hypofluorescence precisely corresponding to the scotoma (Fig. 7d). The hypofluorescent ICGA areas also corresponded to POSL damage (Fig. 8a; arrows). The disease pattern seen with this recurrence clearly corresponded to multifocal choroiditis, therefore a sub-Tenon injection of triamcinolone acetonide (40 mg) was performed considering progression of visual field defects after 4 days and due to the fact that unlike MEWDS, multifocal choroiditis produces scars and carries a high risk of inflammatory choroidal neovascular membranes.

One month after the sub-Tenon’s triamcinolone injection OS, the diffuse peripapillary hypofluorescence recovered on ICGA where only small hypofluorescent dots representing previous chorioretinal scars were still visible (Fig. 7e) and corresponded to POSL reconstitution (Fig. 8b) as well as visual field recovery (Fig. 1, right column). In parallel, microperimetry improved from 328/560 to 424/560. Regular follow-up including ICGA was decided for the patient, as occult choriocapillaritis can occur in the absence of any signs except for ICGA hypofluorescence.

## DISCUSSION

Multiple evanescent white dot syndrome, first described by Lee Jampol et al in 1984, predominantly affects young to middle-aged women; the condition causes unilateral visual loss of variable degrees (severe in some cases) and scotomas objectively identified by visual field testing.^19^–^21^ Characteristic symptoms include photopsia usually reported spontaneously by the patient. The disease usually hits only once and the condition is self limited and favorable with restoration of visual function within 6 to 12 weeks. Funduscopy reveals numerous faint white dots in the mid-periphery at the onset of the disease. These dots can disappear rapidly and may be missed by the clinician if the patient does not present at an early stage; in this case ICGA is especially valuable in reaching a diagnosis.^6^,^22^ ICGA shows numerous hypofluorescent dark areas predominantly in the mid-periphery and around the optic disc where the hypofluorescence is confluent.^8^,^22^ Interestingly, these dark areas are faint in early and intermediate angiographic phases but become much more clearly delineated in the late phase, reflecting choriocapillaris hypo- rather than strict non-perfusion, which probably explains the usually favorable outcome of MEWDS without treatment. ICGA lesions almost integrally resolve spontaneously in the convalescent stage of the disease after 6–12 weeks of evolution. ICGA is the most precise method to monitor disease evolution.^1^

Multifocal choroiditis (MFC) occurs in the same age group as the other PICCPs, namely young to middle aged adults with women being predominantly affected.^23^ Lesions tend to leave scars and most of them are not spontaneously reversible, but active lesions seem to respond to inflammation suppressive therapy. Photopsia is more frequent than in MEWDS and it may be present even when there is no clinical evidence of reactivation. The patients also report scotomas. Multifocal choroiditis can be uni- or bilateral with usually asymmetric involvement.^24^ Perimetry objectively verifies the scotomas reported by the patient. On fundus examination, the typical lesions are small randomly distributed yellow-white atrophic chorioretinal foci in the posterior pole and mid-periphery as well as the periphery. In the active episodes, new lesions are rarely visible on fundus examination and can be very discreet or absent on FA, whereas ICGA is the most sensitive method to detect such occult new lesions.^1^,^25^ One particular feature of multifocal choroiditis is the high proportion of neovascular membranes complicating the disease.^23^

ICGA reveals two sets of signs in MFC. The first type of signs identifies old scarred chorioretinal lesions and consists of hypofluorescent areas persisting up to the late phase, distributed randomly in the fundus, corresponding to hyperfluorescent dots on late FA frames, typical for chorioretinal atrophy from scars of previous inflammatory episodes seen on fundus examination. The second type of signs can be seen along with previously described signs when choroiditis recurs. They consist of hypofluorescent areas, almost silent on FA and not visible on fundus examination, representing areas of new inflammatory involvement. As in MEWDS, some cases of MFC may manifest peripapillary hypofluorescence translating functionally into an enlarged blind spot.^24^ The latter signs respond to systemic corticosteroids and/or immunosuppression and can regress completely if therapy is started early.

Both MEWDS and MFC preferentially affect myopic eyes and have a common denominator, choriocapillaritis.^26^ It is therefore not astonishing that overlap may exist between different entities under the PICCPs described in the past.^14^–^16^ Our patient initially presented with typical features of MEWDS. However, four months after the initial episode of choriocapillaritis, chorioretinal scars appeared which is not typical of MEWDS. Thanks to multimodal imaging, the suspected pathophysiologic disease mechanism could be demonstrated morphologically; ICGA revealed choriocapillaris non-perfusion and spectral domain OCT scans through ICGA hypofluorescent areas demonstrated damage to POSL, a consequence of outer retina ischemia which is oxygenated through the choriocapillaris. FAF showed hyper-autofluorescence in the same ICGA hypofluorescent areas which is interpreted to be due to loss of photoreceptor outer segments^27^. The investigational modality that was best correlated with function (visual fields) was ICGA, since FA and FAF signs regressed later as compared to ICGA signs.

The second episode of choriocapillaritis could not be considered as MEWDS any more since this disease, by definition, occurs only once. Furthermore, additional chorioretinal scars had developed in the meantime, possibly due to silent episodes of choriocapillaritis. The clinical picture seen during the second symptomatic episode of choriocapillaritis corresponded to MFC. The pathophysiologic process was identical to the first episode, showing ICGA non-perfusion and POSL damage which both resolved one month following sub-Tenon’s triamcinolone injection, in parallel to visual field recovery.

It was important to identify the evolution from one PICCP, i.e. MEWDS, to another, i.e. MFC, because the course of MFC can be deleterious and recurrences, best identified by ICGA, have to be managed with inflammation suppressive therapy.

It is however possible that at initial presentation, the patient was already experiencing her first episode of MFC, although it resolved without therapy. The practical attitude to be drawn from this case is, whenever choriocapillaritis/PICCP is suspected, it is important to be aware of the underlying disease mechanism, namely choriocapillaris non-perfusion with outer retinal ischemia at the origin of this group of diseases. An attempt has to be made to classify each case under one of the well-known clinical entities including MEWDS, APMPPE, MFC or serpiginous choroiditis. This however may not be possible, as there are mixed forms, overlapping forms and unclassifiable forms. Therefore, all PICCPs should be followed with ICGA monitoring of choriocapillaris nonperfusion in symptomatic patients. In case of recurrent ICGA hypofluorescence or persistent hypofluorescence in conjunction with functional impairment, inflammation suppressive therapy should be given and evolution monitored using ICGA.

The case described herein demonstrated that FA was less precise than ICGA to delineate the diseased choriocapillaris especially during the second episode. FAF was useful during the first episode showing subclinical involvement, but showed almost no hyper-autofluorescence during the second inflammatory episode. Spectral domain OCT was helpful in delineating lesion morphology in the outer retina explaining functional loss. However, none of these imaging modalities were as closely correlated with functional parameters (visual fields) as ICGA, which represents the best imaging modality to monitor disease evolution.
